# Successful treatment of marrow failure after CARTs for myeloma by the infusion of cryopreserved stem cells

**DOI:** 10.1002/ajh.25664

**Published:** 2019-11-09

**Authors:** Lingzhi Yan, Jingjing Shang, Xiaolan Shi, Huizhu Kang, Wei Liu, Nan Xu, Yong Liu, Guanghua Chen, Liqing Kang, Feiran Gong, Fang Tang, Lei Yu, Depei Wu, Chengcheng Fu

**Affiliations:** ^1^ The First Affiliated Hospital of Soochow University, Jiangsu Institute of Hematology National Clinical Research Center for Hematologic Diseases Suzhou China; ^2^ Institute of Blood and Marrow Transplantation, Collaborative Innovation Center of Hematology, Soochow University Suzhou China; ^3^ Shanghai Unicar‐Therapy Bio‐medicine Technology Co., Ltd Shanghai China; ^4^ Institute of Biomedical Engineering and Technology, Shanghai Engineering Research Center of Molecular Therapeutics and New Drug Development, School of Chemistry and Molecular Engineering, East China Normal University Shanghai China

To the Editor:

1

Chimeric antigen receptor transduced T cell (CART) therapy has demonstrated clinical activity in multiple malignant tumors.^1^ In 2017, the United States Food and Drug Administration approved CD19‐targeted CART cells (CARTs) as a salvage treatment for recurrent and/or refractory patients with B cell malignancies.^2^, ^3^ In recent years, several CART clinical trials for multiple myeloma (MM) conducted worldwide have shown good clinical response, with target antigens including BCMA, CD19, and others.^4^, ^5^, ^6^, ^7^, ^8^, ^9^ In addition to the clinical efficacy of CART therapy, it is important to pay attention to the possible adverse reactions, to reduce the severe, irreversible treatment‐related mortality and to ensure that toxicity is managed well. Prolonged pancytopenia should also be a focus beyond unique acute cytokine release syndrome (CRS).^1^ Such complications may bring the risk of fatal infection and bleeding, and could increase the hospital stay and economic burden of patients. Here, we report one patient with relapsed and refractory MM who developed bone marrow failure and severely prolonged pancytopenia after receiving sequential CD19‐ and BCMA‐specific CARTs. His hematopoiesis was successfully restored by the infusion of cryopreserved autologous stem cells.

Case presentation: A 41‐year‐old male was diagnosed multiple myeloma with IgG lambda in March 2018 after presenting with anemia, mildly elevated creatinine and multiple bone lesions. He received induction therapy with four cycles of bortezomib, thalidomide and dexamethasone (BTD), which resulted in maximum efficacy of partial remission according to the International Myeloma Working Group (IMWG) response criteria.^10^ At this time, the patient was determined to have developed the complication of grade 2 peripheral neuropathy with pain. Autologous stem cells were collected after the administration of high‐dose cyclophosphamide (3 g/m^2^ of body surface area). The harvest in June 2018 contained 7.1 × 10^8^/kg of mononuclear cells and 7.1 × 10^6^/kg of CD34‐positive cells. Unfortunately, the disease progressed during the wait for autologous stem cell transplantation (ASCT). Subsequent second‐line treatment included lenalidomide and dexamethasone (Rd) beginning in July 2018. However, the response was poor, and the disease continued to progress. In September 2018, high‐dose conditional chemotherapy (busulfan 9.6 mg/m^2^ and cyclophosphamide 3.6 g/m^2^) was followed by salvage ASCT. The graft for ASCT was half the amount of the collection. The ASCT resulted in stable disease for 2 months. Considering this poor prognostic finding, the patient was later enrolled in the reported CART trial in our center in December 2017.^11^ A bone marrow aspirate showed weak CD19 expression (0.08%) and strong positive BCMA expression (94.5%) on the clonal plasma cells by flow cytometry. The patient's treatment and administration schedule is shown in Figure 1A.

**Figure 1:**
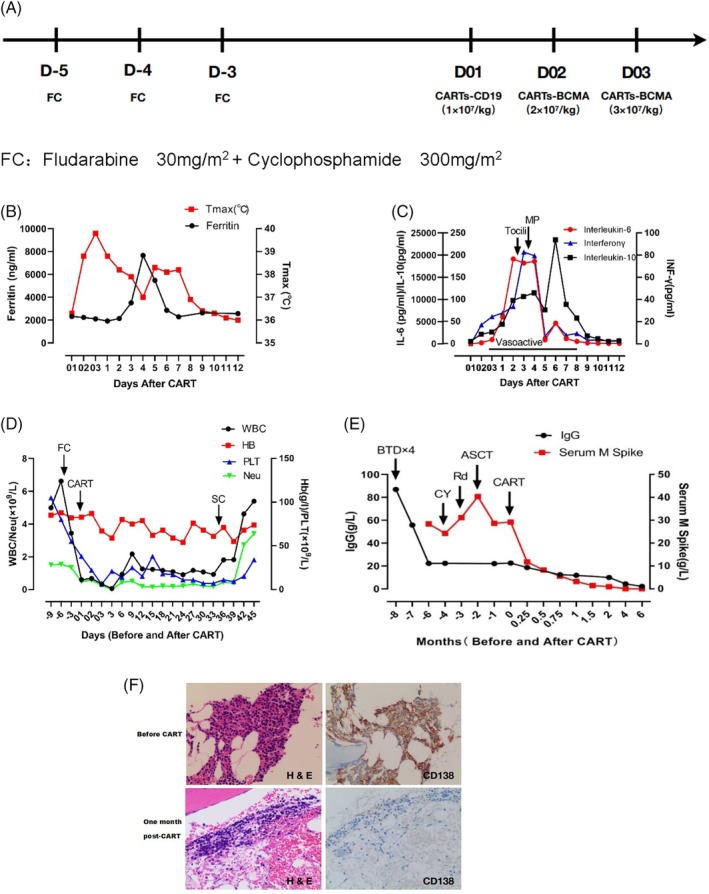
The treatment medication protocol and clinical and laboratory parameters relative to the timing of CART therapy. A, Chemotherapy for lymphocyte depletion included fludarabine and cyclophosphamide. CARTs were infused at a single 1 × 107/kg dose of CD19‐CARTs on day 01 and a split‐dose of BCMA‐CARTs infusion, 40% on day 02 and 60% on day 03 (total 5 × 107/kg dose). B, The patient's temperature rapidly rose post‐CARTs, and his serum ferritin level gradually rose to reach a peak level on day 4 post‐CARTs. The red line represents the patient's maximum temperature in degrees centigrade (°C) per 24‐hour period, with squares demarcating each day. The black line represents the patient's serum ferritin in ng/mL, with circles showing tested values each day. Both parameters returned to baseline on day 9 post‐CARTs. C, The trends of IL‐6, IL‐10 and IFNγ concentrations are shown during the course of CART therapy. The red line represents the patient's serum IL‐6 concentration (pg/ml), with circles showing tested values each day. The black line represents the patient's IL‐10 concentration in pg/mL with squares representing the tested values each day. The blue line represents the patient's IFNγ concentration in pg/mL, with triangles showing the tested values each day. Time on vasoactive medications (norepinephrine) is indicated by a black line under “vasoactives” (days 03 to 8). D, The trends of blood cell count are exhibited during and after CART treatment. The black, red, blue and green lines respectively represent the levels of white blood cells, hemoglobin, platelets and neutrophils. The initiation of each treatment regimen is depicted by an arrow. E, IgG and serum M protein levels are shown throughout the treatment course. The initiation of each treatment regimen is depicted by an arrow. F, Hematoxylin and eosin staining and immunohistochemical (IHC) staining for CD138. Bone marrow cells were 50% clonal plasma cells, as shown by CD138 staining before CARTs infusion (original magnification, × 100). Bone marrow examination showed severe aplastic cellularity, and no plasma cells could be detected 1 month post‐CARTs

The patient presented a fever of 38.8°C ten hours after infusion of the first dose of CARTs‐CD19, and the peak of temperature was 39.8°C on day two, and lasted for a total of 9 days. The peak serum ferritin level was also nearly four times higher compared to the baseline (Figure 1B). Peak serum interleukin (IL)‐6, IL‐10 and interferon‐γ levels were detected on day two postinfusion (Figure 1C). According to the guidelines of the CARTOX Working Group,^12^ the patient had developed grade 3 CRS at this time. We treated the patient with a single 6 mg/kg dose of the IL‐6R inhibitor tocilizumab. However, he continued to clinically deteriorate and experienced hypoxemia grade 1, elevated creatinine grade 2, elevated bilirubin grade 2,^13^ and hypotension requiring support with a 0.40‐0.45 μg/kg/min drop rate of norepinephrine. This toxicity culminated on day three post CART therapy. Methylprednisolone 2 mg/kg was administered to minimize the toxicity and to improve the patient's clinical symptoms. There was no neurologic toxicity. After IL‐6R antibody and steroid administration, his cytokine levels, organ functions and clinical manifestations were dramatically ameliorated. On day eight postinfusion, we were able to discontinue the norepinephrine infusion (Figure 1C).

However, the patient exhibited long‐term grade 4 hematological toxicity after CART treatment. The onset of pancytopenia occured after fludarabine and cyclophosphamide regimen for lymphocyte depletion. The patient presented only with severe pancytopenia without any other manifestations, such as fever or organ damage. Granulocyte colony‐stimulating factor and thrombopoietin support did not work after the CRS reaction was completely subsided. The patient was platelet‐ and red blood cell transfusion‐dependent from day two until more than 30 days after CARTs infusion. The gene mutations related to hemophagocytic lymphohistiocytosis testing, such as LYST, PRF1, CD27, RAB27A, BLOC1S6, UNC13D, STXBP2, TCN2, AP3B1, ITK, STX11, SH2D1A, XIAP and MAGT1, were normal. Parvovirus B19 and cytomegalovirus were negative. Bone marrow aspirate and biopsy specimens demonstrated extreme aplastic bone marrow (<5% cellularity) with no megakaryocytes and reticulin fibrosis at 1 month postinfusion. On the 35th day post‐CARTs, autologous cryopreserved stem cells, containing 3.05*10^6^/kg CD34‐positive cells, were infused considering the limited value of tandem ASCT. As expected, the patient's hematopoietic function was gradually reconstructed on the 11th day after infusion of autologous stem cells (Figure 1D).

The trends of the patient's serum IgG concentration and the serum monoclonal protein concentration (M spike) throughout the treatment are shown in Figure 1E. The serum monoclonal protein became undetectable 4 months postinfusion and remained undetectable until July 2019. After CART therapy, bone marrow aspirate and biopsy also confirmed that the patient achieved stringent complete remission (Figure 1F), including minimal residual disease negative by flow cytometry. The laboratory characteristics of the patient's bone marrow before and after CART treatment are shown in Table 1. The CARTs maximum amplified 16.34‐fold on day eight postinfusion, and sustained for 29 days by quantitative PCR using a transgene‐specific primer/probe pair.

**Table 1:** Laboratory characteristics of the patient's bone marrow before and after CART treatment

	Before CARTs			After CARTs			
	At diagnosis	Before ASCT	After ASCT	1 month	2 months	3 months	6 months
Cellularity	packed	hypercellular	hypercellular	aplastic	hypercellular	normocellular	normocellular
Clonal PC^a^(%)	95	60	50	absent	absent	absent	absent
FISH^b^	negative	negative	negative	negative	negative	negative	negative
MRD^c^	NA^d^	positive	positive	negative	negative	negative	negative

a PC, plasma cell.

b FISH, fluorescence in situ hybridization. FISH probe covers the chromosome region including 13q14 (Rb1), 1q21, 14q32 (IgH) and 17p13(P53) in this study.

c MRD, minimal residual disease using ten‐color flow cytometry.

d NA, not applicable.

Note, CART therapy is associated with unique CRS toxicities. Higher peak in vivo proliferation of CARTs is possibly associated with a clinical response, but it has also been associated with CRS grade.^3^, ^14^, ^15^ Thus, CRS is an inflammatory syndrome caused by multiple cytokines produced by the CARTs themselves and by other cells, and can induce damage to multiple systems, including the hematopoietic system.

Multiple hematologic toxicities may occur following CARTs infusion, including the development of anemia, thrombocytopenia and neutropenia. Prolonged cytopenias have been previously reported in limited clinical trials.^2^, ^7^ However, the extent of suppression and effective management measures have not been described in detail. Conditioning chemotherapy may contribute to the development of pancytopenia. However, this effect can generally be restored within 1 to 2 weeks. In this trial, the CARTs targeting CD19 may have resulted in CAR‐mediated damage to normal B lymphocytes, but this effect did not explain the suppression of multiple hematopoietic series in the patient. Cell‐stimulating factor support did not improve the blood test results. The biochemical parameters and elevated ferritin profile did not fulfill the hemophagocytic lymphohistiocytosis diagnostic criteria.^12^ Therefore, the adverse event probably had other causes. We determined that the patient's bone marrow hematopoiesis was in a state of exhaustion and failure. His serum IL‐6 level increased more than 3000‐fold, and his IFNγ level also increased dozen‐fold compared to the patient's baseline levels. This study demonstrated that CART treatment may have caused some damage to early hematopoietic cells, including hematopoietic stem/progenitor cells. Considering that CARTs targeting CD19 and BCMA probably have no direct damage to normal early hematopoietic cells, we speculate that this result is most likely due to a large amount of inflammatory cytokine release.

In summary, our work demonstrated that autologous stem cell infusion may be considered in cases of life‐threatening pancytopenia post‐CART. Seriously prolonged pancytopenia may also emerge in more CART therapy clinical practices. Research priorities include achieving a better mechanistic understanding the reasons for the cytopenia observed after CART therapy. This patient's inflammatory factors had returned to the baseline level and had no significant expansion of CARTs in vivo when receiving stem cell infusion. Improved animal models and CART clinical trial data will likely provide more evidence for addressing this question, and further determining the time of intervention for stem cell infusion.

## CONFLICT OF INTEREST

All authors declare that they have no conflicts of interest. The research protocol referenced in this manuscript has been approved by the Ethics Committee of the First Affiliated Hospital of Soochow University and followed the tenets of the Helsinki Declaration of 1975, as revised in 2000 (5). Informed consent was obtained from the patient in the study.

## AUTHOR CONTRIBUTIONS

L.Y., D.W. and C.F planned the trial and experiments; L.Y., J.S., X.S., H.K., W.L., N.X., Y.L., G.C., L.K. and F.G. conducted experiments; L.Y., J.S. and C.F. analyzed the data; L.Y. and C.F wrote the manuscript; and L.Y., J.S., X.S., H.K., F.T. and C.F. provided patient care. Conflict of interest disclosure: L.Y. has patent applications for anti‐CD19 and anti‐BCMA CARs.
